# Single-Shot Subcutaneous Lidocaine Infiltration at Closure Is Associated with Reduced Early Pain and Opioid Requirement After Single-Incision Laparoscopic Totally Extraperitoneal Hernia Repair

**DOI:** 10.3390/jcm14238324

**Published:** 2025-11-23

**Authors:** Jong Min Lee

**Affiliations:** Department of Surgery, Yongin Severance Hospital, Yonsei University College of Medicine, Yongin 16995, Republic of Korea; hiposoath@yuhs.ac; Tel.: +82-31-5189-8467; Fax: +82-31-5189-8566

**Keywords:** SILTEP, lidocaine infiltration, inguinal hernia repair, postoperative pain, opioid-sparing analgesia, propensity score matching

## Abstract

**Background:** Subcutaneous wound infiltration with local anesthetics has been proposed as a simple adjunct for postoperative pain control; however, its value in single-incision laparoscopic total extraperitoneal (SILTEP) inguinal hernia repair remains unclear. **Methods:** We retrospectively analyzed 199 consecutive SILTEP inguinal hernia repairs performed between November 2022 and July 2025 (117 no-lidocaine, 82 lidocaine). A double adjustment, combining 1:1 propensity score matching with multivariable regression across 20 multiply imputed datasets was performed. The primary outcome was maximal numeric pain intensity scale (NPIS) on postoperative day (POD) 0. **Results:** Eighty-two matched pairs were generated. In the final pooled, adjusted models, lidocaine infiltration was associated with a significant reduction in the primary outcome, maximal NPIS on POD 0 (β = −1.25; 95% CI: −2.01 to −0.50; *p* = 0.001). Lidocaine was also associated with significantly lower odds of requiring rescue analgesia on POD 0 (OR = 0.12; 95% CI: 0.03–0.46; *p* = 0.002), fewer rescue doses during hospitalization (β = −1.11; 95% CI: −1.62 to −0.49; *p* < 0.001), and a lower morphine-equivalent dose (β = −5.14; 95% CI: −7.79 to −2.49; *p* < 0.001). No increase in postoperative complications was observed. **Conclusions:** Single-shot subcutaneous lidocaine infiltration in SILTEP hernia repair is a simple, low-risk intervention that was associated with reduced immediate postoperative pain and opioid use without increasing complications. However, the effect was short-lived with no sustained benefit beyond the first postoperative day.

## 1. Introduction

Inguinal hernia repair is among the most frequently performed surgical procedures worldwide [1]. Compared with open repair, laparoscopic approaches are associated with less early postoperative pain and faster recovery [2]. Building on this minimally invasive paradigm, single-incision laparoscopic total extraperitoneal (SILTEP) repair has emerged as a feasible alternative to multiport laparoscopy. Early reports suggest that SILTEP offers improved cosmesis and may accelerate recovery, thereby extending the advantages of minimally invasive surgery [3,4].

Despite these advances, early postoperative pain after laparoscopic inguinal hernia repair remains clinically relevant and can delay recovery or discharge in some patients. In ambulatory pathways, inadequate pain control is a leading cause of unplanned overnight admission or subsequent healthcare utilization, highlighting the need for effective, opioid-sparing multimodal analgesia consistent with enhanced recovery after surgery (ERAS) principles [5,6,7]. Regional abdominal wall blocks, such as ultrasound-guided transversus abdominis plane (TAP) and quadratus lumborum blocks (QLB), can reduce early pain and opioid requirements; however, they require ultrasound equipment, technical expertise, and additional time, which may limit their adoption in fast-track, day-case settings [8,9].

In contrast, local anesthetic infiltration at trocar or wound sites is simple, reproducible, and readily integrated into routine surgical workflows. Across a range of laparoscopic procedures, wound or port-site infiltration has been reported to reduce early postoperative pain and, in some studies, to decrease opioid consumption [10,11]. However, most evidence comes from conventional multiport laparoscopy or open surgery [12], and data specific to single-incision repairs remain scarce. Whether infiltration provides a measurable analgesic benefit after SILTEP repair remains unclear.

We aimed to evaluate the effects of subcutaneous lidocaine infiltration on early postoperative pain in patients undergoing SILTEP hernia repair. As laparoscopic repair produces less baseline pain than open surgery, even a modest additional reduction could be clinically meaningful, facilitating earlier discharge and further supporting ERAS objectives.

## 2. Materials and Methods

### 2.1. Study Design and Patients

We conducted a retrospective analysis of a prospectively maintained database of consecutive patients who underwent SILTEP repair for inguinal hernia by a single surgeon (J.M.L.) at Yongin Severance Hospital between November 2022 and July 2025. After obtaining formal approval from the Institutional Review Board (IRB) of Yongin Severance Hospital (Project No. 9-2025-0138; 29 September 2025), this existing dataset was analyzed. Adults (≥18 years) with symptomatic inguinal hernias were considered candidates for SILTEP. Patients requiring emergency surgery (e.g., for bowel incarceration or strangulation) and those in whom preperitoneal dissection was expected to be difficult (e.g., after prior pelvic surgery such as prostatectomy) were generally not considered for this approach.

**Figure 1:**
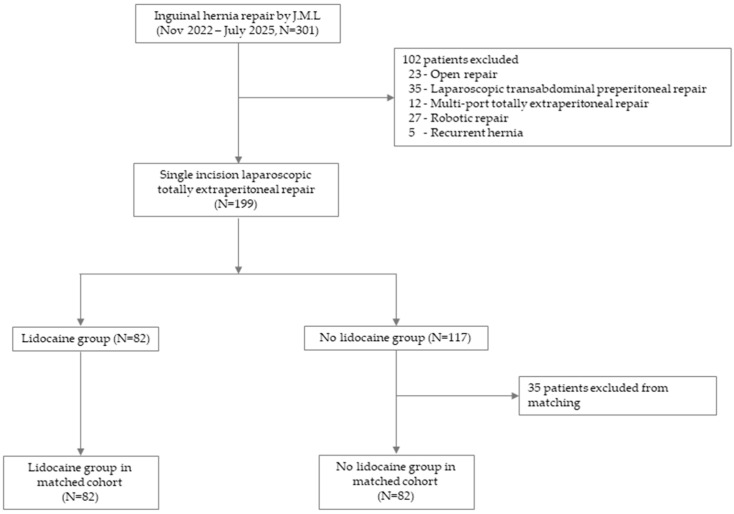
Flow chart of patient selection.

Overall, 301 inguinal hernia surgeries were performed during the study period. Cases managed with open repair (n = 23), laparoscopic transabdominal preperitoneal repair (n = 35), multi-port totally extraperitoneal (TEP) repair (n = 12), robotic repair (n = 27), or recurrent hernia repair (n = 5) were excluded, leaving 199 primary SILTEP cases for analysis. Exposure groups were defined on an as-treated basis according to operative records. Subcutaneous lidocaine infiltration was adopted as the surgeon’s routine practice from November 2024 onward. Therefore, the two groups corresponded to procedures performed before and after this change in standard practice. Patients who received subcutaneous lidocaine infiltration at wound closure comprised the lidocaine group (n = 82), and those who did not comprise the non-lidocaine group (n = 117) (Figure 1).

### 2.2. Surgical Technique and Lidocaine Infiltration Protocol

The operative setup and SILTEP technique have been described previously [13]. In brief, a single 2.0–2.5 cm intraumbilical incision was made, and the preperitoneal working space was bluntly created without a balloon dissector. The indirect hernia sac was dissected and ligated with an absorbable loop, and peritonealization of the sac was routinely performed. A lightweight mesh of an appropriate size was introduced into the preperitoneal space to cover the entire myopectineal orifice. Fibrin sealant was applied over the mesh, and tacker fixation was avoided unless clinically indicated. The anterior rectus sheath was closed using a 2-0 absorbable suture.

For patients in the lidocaine group, a 1.8 mL cartridge of 2% lidocaine hydrochloride with epinephrine 1:100,000 (36 mg lidocaine HCl and 18 μg epinephrine base, equivalent to 32.4 μg epinephrine bitartrate; Huons Co., Seongnam, Republic of Korea) was diluted with normal saline to a total volume of 10 mL. Immediately before skin closure, this solution was infiltrated into the subcutaneous tissue along the umbilical incision in four evenly spaced 2.5 mL aliquots. The skin and subcutaneous layers were subsequently closed using 3-0 absorbable sutures.

### 2.3. Intraoperative Anesthesia and PACU Management

All anesthetic and analgesic management described below reflected routine clinical practice at our institution, and no procedures were modified for the purposes of this retrospective study. General anesthesia was administered by the attending anesthesiologists according to institutional protocols. Standard monitors (electrocardiography, non-invasive blood pressure, pulse oximetry, capnography, and temperature) were used. After preoxygenation, anesthesia was induced with propofol and a short-acting opioid (fentanyl bolus or remifentanil infusion) at the anesthesiologist’s discretion, and tracheal intubation was facilitated with a non-depolarizing neuromuscular blocker. Ventilation was adjusted to maintain normocapnia, and anesthesia was titrated to routine hemodynamic targets. Hemodynamic fluctuations were managed with small doses of vasoactive agents as required. Neuromuscular blockade was reversed, patients were extubated upon meeting the standard criteria, and then transferred to the post-anesthesia care unit (PACU).

In the PACU, all patients underwent routine hemodynamic monitoring and regular assessment of consciousness and pain levels. Opioids were selectively administered at the discretion of the anesthesiologist. When indicated, fentanyl was administered either as intermittent boluses (25–50 μg) or as a brief infusion on arrival. Non-opioid analgesics (intravenous acetaminophen, ketorolac, or nefopam) were administered near the end of surgery or in the PACU, as clinically indicated. Ultrasound-guided abdominal wall block was not performed during the study period. Apart from the subcutaneous lidocaine infiltration, no additional local anesthetic infiltration was undertaken.

### 2.4. Postoperative Ward Management

In the immediate postoperative period, patients were allowed sips of water when fully awake and advanced to a soft diet on postoperative day (POD) 0, as tolerated. Intravenous crystalloids were continued as per routine order, and early ambulation was encouraged. Oral combination analgesia (acetaminophen 250 mg, codeine phosphate 10 mg, and ibuprofen 200 mg per tablet; Mypol^®^, Sungwon Adcock Pharmaceutical Co., Ltd., Hwaseong, Republic of Korea) was initially used as the standard regimen, but in some cases was later replaced by acetaminophen alone due to adverse effects (e.g., postoperative nausea, dizziness) or contraindications in patients with chronic kidney disease. This oral regimen was prescribed at discharge, typically as a 5-day supply. In the ward, as-needed parenteral analgesia for breakthrough pain included tramadol 50 mg IV (a second 50 mg dose permitted if pain persisted) or, if inadequate, meperidine 25 mg IM at the physician’s discretion. No postoperative patient-controlled analgesia was administered. The patients were discharged on POD 1 and returned for an outpatient review on POD 7.

### 2.5. Measurement of Postoperative Pain Intensity

Postoperative pain was assessed using the Wong–Baker Faces Pain Rating Scale (WBFPRS) in the PACU and the 11-point numeric rating scale (NPIS, 0–10; 0 = no pain, 10 = worst imaginable pain) according to institutional nursing protocols. Immediately after transfer from the PACU to the ward, patients underwent routine pain assessment. During ongoing treatment with scheduled analgesics (e.g., intravenous injections or oral analgesics), pain was evaluated twice daily, typically once during the day shift and once in the evening, and no separate assessments were performed at night. When patients reported pain requiring additional (PRN) analgesia, the NPIS scores were recorded before administration and re-evaluated 30 min after parenteral analgesic administration or 1 h after oral analgesic administration. Patients who consistently reported no pain (NPIS score = 0) generally underwent a single daily assessment during the day shift. The value closest to the nominal time point was used when multiple scores were available within the same time window. Missing time points were not imputed.

### 2.6. Outcomes and Variable Definitions

The primary exposure variable was the receipt of subcutaneous lidocaine infiltration (lidocaine group vs. no-lidocaine group). Postoperative pain was assessed using the WBFPRS in the PACU and the 11-point numeric rating scale on the ward, according to institutional nursing protocols. While the scales are not identical, both are validated ordinal measures for acute postoperative pain with comparable 0–10 scoring systems. Therefore, WBSPRS were treated as direct equivalents to NPIS 0–10 scores for this analysis.

The study outcomes were defined accordingly:

Primary outcome: The primary outcome was the maximal NPIS on POD 0. This endpoint was selected as it represents the most intense pain experienced on the day of surgery, which is the period of highest clinical relevance for an acute pain intervention and the main driver for rescue analgesic requirements.

Secondary outcomes: Incidence of rescue analgesia on POD 0, the total number of rescue doses during hospitalization, and total postoperative morphine equivalent dose (MED), Individual NPIS scores at specified time points (0, 0.5, 1, 2, 4, 8, 12, and 24 h) and maximal NPIS on POD 1. Length of stay (LOS) and postoperative complications (e.g., seroma, surgical site infection [SSI], acute urinary retention [AUR]).

All parenteral analgesics administered postoperatively were converted to a MED. The specific conversion factors, based on ENCORE study [14] guidelines, are detailed in Supplementary Table S1. Nefopam was not listed in these guidelines and was therefore not included in the MED calculation. Its use was captured as a categorical variable.

### 2.7. Statistical Analyses

Analyses were conducted on an as-treated basis (lidocaine versus non-lidocaine). Continuous variables were tested for normality using the Shapiro–Wilk test and summarized as mean ± SD or median [Q1–Q3], whereas categorical variables were summarized as counts (%). Between-group comparisons were performed with Welch’s t test or the Wilcoxon rank-sum test for continuous variables, and with the χ^2^ test or Fisher’s exact test for categorical variables, as appropriate.

To address potential confounding factors, the propensity score for receiving subcutaneous lidocaine infiltration was estimated by logistic regression, including pre-specified covariates: age, sex, bilateral hernia, European Hernia Society (EHS) groin hernia size class, BMI ≥ 25 kg/m^2^, prior lower abdominal surgery, American Society of Anesthesiologists Physical Status (ASA-PS) ≥ III, treatment period, and intraoperative use of IV opioids, non-opioid analgesics, dexamethasone, and lidocaine. Missing values were handled using multiple imputation (M = 20) with predictive mean matching. The imputation model included the treatment indicator, all covariates, and all postoperative outcome variables.

1:1 propensity score matching (PSM) (optimal method) was then performed within each of the 20 imputed datasets. Covariate balance before and after matching was assessed using standardized mean differences (SMDs), with |SMD| < 0.1 considered adequate balance. Post-matching analyses were conducted for the matched cohort using the aforementioned statistical tests. Where applicable, multivariable linear or logistic regression was performed, reporting β coefficients or odds ratios (ORs) with 95% confidence intervals (CIs). All final regression analyses were conducted by pooling the results from these 20 datasets using Rubin’s rules.

A two-sided *p* < 0.05 was considered statistically significant for the primary outcome (maximal NPIS on POD 0) and all other comparisons. To address multiplicity for the nine secondary NPIS-related outcomes, the significance threshold was set at *p* < 0.0056 (0.05/9) based on the Bonferroni correction. E-values were computed for the primary pain-specific outcome to evaluate the robustness of the association to potential unmeasured confounding.

Analyses were performed using R (version 4.5.1; R Foundation for Statistical Computing, Vienna, Austria). Core statistical functions, including linear and logistic regression analyses, were implemented using the *stats* package. PSM was conducted with *MatchIt* (version 4.7.2), and multiple imputation was performed with *mice* (version 3.18.0). Covariate balance before and after matching was assessed and visualized using *cobalt* (version 4.6.1). The C-statistic (area under the receiver operating characteristic curve, AUC) for the propensity score model validation was calculated using *pROC* (version 1.19.0.1). The E-value was computed using the *EValue* (version 4.1.4) package.

## 3. Results

### 3.1. Baseline Characteristics Before and After Matching

Among the 199 patients (117 without lidocaine; 82 with lidocaine), the lidocaine group had higher proportions of ASA-PS ≥ III, bilateral hernia, and intraoperative IV lidocaine use before matching (Table 1). After 1:1 optimal matching, 82 patient pairs were identified. Covariate balance was evaluated using the first imputed matched dataset, which is presented as a representative example in Table 1 (corresponding to the first of 20 imputed datasets) and visualized in Supplementary Figure S1.

Matching achieved balance for most baseline variables. However, treatment period (post-matching SMD = 2.18), intraoperative IV lidocaine use (SMD = 0.21), intraoperative IV opioid use (SMD = 0.15), and EHS size classification (SMD = 0.11) remained imbalanced.

The discriminative ability of the propensity score model was excellent, with a C-statistic (AUC) of 0.92 for the first imputed dataset.

### 3.2. Postoperative Outcomes

In the overall cohort, patients in the lidocaine group had lower frequencies of any rescue analgesia, rescue analgesia on postoperative day (POD) 0, number of rescue doses, and morphine-equivalent doses than those in the no-lidocaine group (Table 2). These differences in the matched cohort (the first imputed matched dataset) remained for the mean number of rescue doses (0.46 ± 0.65 vs. 0.96 ± 1.52; *p* = 0.007) and morphine-equivalent dose [0.0 (0.0–5.0) vs. 5.0 (0.0–5.0); *p* = 0.028]. The frequencies of any rescue analgesia (51.2% vs. 37.8%; *p* = 0.116) and rescue analgesia on POD 0 (48.8% vs. 34.1%; *p* = 0.081) were no longer statistically significant. Other postoperative outcomes were comparable between the groups in both overall and matched cohorts.

**Figure 2:**
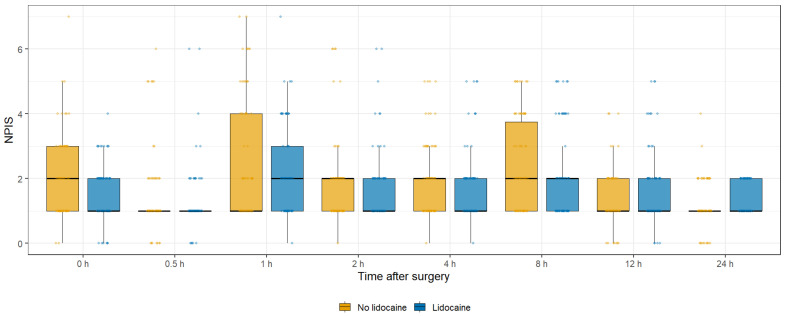
Time course of postoperative NPIS in matched patients undergoing SILTEP repair with or without subcutaneous lidocaine infiltration. Data are displayed as boxplots; the thick horizontal black line within each box represents the median, the box edges indicate the interquartile range, and the whiskers show the minimum and maximum values excluding outliers.

**Figure 3:**
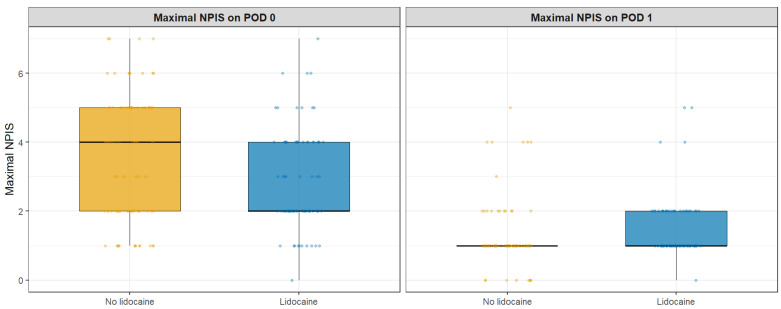
Maximal NPIS on POD 0 (primary outcome, left panel) and POD 1 (secondary outcome, right panel) in matched patients undergoing SILTEP repair with or without subcutaneous lidocaine infiltration. Data are displayed as boxplots; the thick horizontal black line within each box represents the median, the box edges indicate the interquartile range, and the whiskers show the minimum and maximum values excluding outliers.

In the matched cohort, the primary outcome—maximal NPIS on POD 0—was lower in the lidocaine group [2 (2–4) vs. 4 (2–5); *p* = 0.023]. Among secondary pain outcomes, several showed nominal differences (e.g., NPIS at 24 h [1 (1–1) vs. 1 (1–2); *p* = 0.010] and maximal NPIS on POD 1 [1 (1–1) vs. 1 (1–2); *p* = 0.018]), but none met the Bonferroni-adjusted significance threshold (α = 0.0056) (Table 3, Figure 2 and Figure 3, Supplementary Table S2).

### 3.3. Post-Matching Regression Adjustment: Pain-Related Outcomes

Multivariable regression models based on pooled results identified a significant reduction in the primary outcome, maximal NPIS on POD 0 (β = −1.25; 95% CI, −2.01 to −0.50; *p* = 0.001) (Table 4). Lidocaine infiltration was also associated with fewer rescue doses (β = −1.11; 95% CI, −1.62 to −0.49; *p* < 0.001), a lower morphine-equivalent dose (β = −5.14; 95% CI, −7.79 to −2.49; *p* < 0.001), and lower NPIS at 0 h (β = −0.81; 95% CI, −1.27 to −0.35; *p* < 0.001). The E-value for maximal NPIS on POD 0 was 3.32 (CI limit 1.60).

Rescue analgesia on POD 0, lidocaine infiltration was associated with reduced odds of requiring rescue medication (OR = 0.12; 95% CI, 0.03–0.46; *p* = 0.002). The corresponding absolute risk reduction (ARR) was 17.9% (95% CI, 2.3–33.6%), yielding a number needed to treat (NNT) of 5.6 (95% CI, 3.0–44.1). The E-value for rescue analgesia on POD 0 was 16.1. Conversely, NPIS at 24 h was slightly higher in the lidocaine group (β = 0.26; 95% CI, −0.01 to 0.53; *p* = 0.062), but this difference was not statistically significant after Bonferroni correction.

## 4. Discussion

Our study found that subcutaneous lidocaine infiltration was associated with lower immediate postoperative pain intensity and a reduced need for rescue analgesia after SILTEP repair, with no increase in postoperative complications compared with the non-lidocaine group. To the best of our knowledge, limited evidence has specifically examined the impact of local anesthetic infiltration on pain outcomes after SILTEP repair.

Intraoperative local wound infiltration has been investigated across a variety of surgical procedures; however, reported outcomes remain inconsistent. Gami et al. [12] demonstrated that subcutaneous bupivacaine with epinephrine reduced 24 h pain scores (visual analog scale 2.41 vs. 2.21) and lowered the proportion of patients requiring rescue opioids (25.5% vs. 13.3%) after cesarean delivery. In single-incision laparoscopic appendectomy [10], incisional bupivacaine was associated with lower pain scores at 1, 6, and 12 h, along with fewer additional analgesic administrations (1.2 ± 0.5 vs. 0.2 ± 0.2 times). However, in multiport laparoscopic totally extraperitoneal (TEP) repair, Kulasegaran et al. [15] reported that pre-peritoneal infiltration of 0.25% bupivacaine with epinephrine did not improve pain scores at 4 or 24 h compared to controls. More recently, Jain et al. [16] found that ropivacaine infiltration into port sites and the pre-peritoneal space failed to reduce postoperative pain or analgesic requirements in TEP repair. Collectively, these findings suggest that the effectiveness of local wound infiltration depends on the surgical procedure, anesthetic agent, and plane of administration.

In our study, subcutaneous lidocaine infiltration resulted in a 1.25-point lower adjusted mean for the primary outcome, maximal NPIS on POD 0 (β = −1.25; 95% CI, −2.01 to −0.50), which represents a clinically meaningful reduction in early postoperative pain [17]. In addition, the ARR for rescue analgesia on POD 0 was 17.9% (NNT = 5.6), indicating that one in every six patients receiving lidocaine infiltration would avoid the need for rescue medication. After fascia closure, lidocaine infiltration directly targets the superficial nerves responsible for somatic wound pain. This approach allows the direct visualization of the injection plane and minimizes the risk of vascular injury or deep hematoma. Subcutaneous tissue generally has a lower density than visceral or perivascular fat [18], with reduced vascularity and perfusion in this compartment, which may prolong local retention of infiltrated anesthetics. In contrast, the preperitoneal and intramuscular planes are more vascularized and diffuse, promoting faster systemic absorption and diminishing local effects [19]. Given that most postoperative pain after laparoscopic hernia repair arises from the periumbilical site where fascial closure is performed, subcutaneous infiltration at the single-incisional wound is an appropriate choice that provides a measurable analgesic effect.

We selected lidocaine over longer-acting agents such as bupivacaine because of its rapid onset, broad clinical use, and favorable cardiac safety profile. Lidocaine typically achieves anesthesia within minutes but lasts only 1–2 h in the tissue, aligning with our finding that its analgesic benefit wanes by 12–24 h [20]. While bupivacaine offers a longer duration of action—up to 4–6 h with epinephrine—it has a higher cardiotoxic potential because of its slow dissociation from cardiac sodium channels, increasing the risk of inadvertent intravascular injection [21,22]. In our clinical setting, lidocaine was readily available, easy to dose safely, and did not cause any agent-related complications. Thus, it serves as an effective and safe initial strategy, with the potential for future protocols to enhance outcomes using longer-acting local anesthetics.

This study has several important limitations. First, as a single-center study involving only a single surgeon, our findings may have limited generalizability and may not be applicable to other institutions or surgeons with different baseline protocols or surgical techniques. Furthermore, this non-randomized study remains subject to residual confounding, despite the use of PSM and post-matching regression. However, the E-values for maximal NPIS on POD 0 (3.32) and rescue analgesia on POD 0 (16.1) suggest that only a relatively strong unmeasured confounder could explain away the observed associations, supporting the robustness of our findings. Changes in routine systemic analgesia during the study period may have introduced treatment-era effects that are difficult to distinguish from the impact of the infiltration strategy. Pain assessment was limited to NPIS at rest within 24 h, without evaluation of long-term outcomes such as activity-related pain, return to function, chronic pain, patient satisfaction, or opioid-related adverse events. Finally, because the intervention involved plain lidocaine, conclusions regarding analgesic duration and 24 h findings may not be generalizable to longer-acting agents or continuous wound infiltration techniques.

## 5. Conclusions

In conclusion, for SILTEP hernia repair, single-shot subcutaneous lidocaine infiltration is a simple, low-risk intervention that was associated with measurable early analgesic benefits and reduced opioid use on the day of surgery, without any increase in drug-related complications. However, the effect was short-lived with no sustained benefit beyond the first postoperative day. Future randomized trials that standardize systemic analgesia and directly compare lidocaine with longer-acting local anesthetics are warranted to establish an optimal multimodal strategy for achieving pain-free recovery after laparoscopic hernia repair.

## Figures and Tables

**Table 1:** Baseline characteristics.

	Overall Cohort			PSM Cohort		
	No Lidocaine (n = 117)	Lidocaine (n = 82)	SMD	No Lidocaine (n = 82)	Lidocaine (n = 82)	SMD
Age	62.9 ± 14.8	65.0 ± 17.2	0.13	64.0 ± 14.2	65.0 ± 17.2	0.06
Male sex	107 (91.5)	80 (97.6)	0.27	80 (97.6)	80 (97.6)	<0.01
BMI > 25 kg/m^2^	40 (34.2)	21 (25.6)	0.19	24 (29.3)	21 (25.6)	0.08
ASA PS ≥ III	36 (30.8)	37 (45.1)	0.30 *	33 (40.2)	37 (45.1)	0.09
Previous lower abdominal surgery	25 (21.4)	16 (19.5)	0.05	17 (20.7)	16 (19.5)	0.03
Bilateral hernia	19 (16.2)	5 (6.1)	0.33 *	6 (7.3)	5 (6.1)	0.05
EHS size classification			0.24			0.11
1	39 (33.9)	19 (23.2)		23 (28.0)	19 (23.2)	
2	68 (59.1)	56 (68.3)		53 (64.6)	56 (68.3)	
3	8 (7.0)	8 (8.5)		6 (7.3)	7 (8.5)	
Treatment period			2.35			2.18
2022.11–2024.10	111 (94.9)	16 (19.5)		76 (92.7)	16 (19.5)	
2024.11–2025.07	6 (5.1)	66 (80.5)		6 (7.3)	66 (80.5)	
Intraoperative IV analgesia use						
Opioid	31 (26.5)	32 (39.0)	0.27	26 (31.7)	32 (39.0)	0.15
Non-opioid	67 (57.3)	47 (57.3)	<0.01	50 (61.0)	47 (57.3)	0.07
Dexamethasone	49 (41.9)	36 (43.9)	0.04	35 (42.7)	36 (43.9)	0.02
Lidocaine	108 (92.3)	67 (81.7)	0.32 *	73 (89.0)	67 (81.7)	0.21

Data are expressed as n (%) or mean ± standard deviation. An asterisk (*) indicates variables with *p* < 0.05 in univariate comparisons. Abbreviations: SMD, standardized mean difference; BMI, body mass index; ASA PS, American Society of Anesthesiologists Physical Status; EHS, European Hernia Society; IV, intravenous.

**Table 2:** Postoperative use of analgesia and complications.

	Overall Cohort			PSM Cohort		
	No Lidocaine (n = 117)	Lidocaine (n = 82)	*p*	No Lidocaine (n = 82)	Lidocaine (n = 82)	*p*
Any rescue analgesia	68 (58.1)	31 (37.8)	0.007	42 (51.2)	31 (37.8)	0.116
Rescue analgesia on POD 0	66 (56.4)	28 (34.1)	0.003	40 (48.8)	28 (34.1)	0.081
Rescue analgesia on POD 1	9 (7.7)	8 (9.8)	0.799	6 (7.3)	8 (9.8)	0.780
Number of rescue doses during hospitalization	1.00 ± 1.36	0.46 ± 0.65	0.008	0.96 ± 1.52	0.46 ± 0.65	0.007
Morphine equivalent dose (mg)	5.0 (0.0–5.0)	0.0 (0.0–5.0)	0.002	5.0 (0.0–5.0)	0.0 (0.0–5.0)	0.028
Additional analgesia at outpatient follow-up	4 (3.4)	1 (1.2)	0.329	4 (4.9)	1 (1.2)	0.364
LOS	1.0 (1.0–1.0)	1.0 (1.0–1.0)	0.900	1 (1–1)	1 (1–1)	0.855
All complications	8 (6.8)	5 (6.1)	0.835	5 (6.1)	5 (6.1)	>0.999
Seroma	4 (3.4)	6 (7.3)	0.215	3 (3.7)	6 (7.3)	0.493
SSI	4 (3.4)	1 (1.2)	0.329	2 (2.4)	1 (1.2)	>0.999
AUR	3 (2.6)	0 (0.0)	0.144	1 (1.2)	0 (0.0)	>0.999
Arrhythmia	1 (0.9)	0 (0.0)	>0.999	1 (1.2)	0 (0.0)	>0.999

Data are expressed as n (%), mean ± standard deviation, or median (interquartile range), as appropriate. Abbreviations: POD, postoperative day; LOS, length of stay; SSI, surgical site infection; AUR, acute urinary retention.

**Table 3:** Postoperative pain intensity in the PSM cohort.

	No Lidocaine (n = 82)	Lidocaine (n = 82)	*p*
Primary outcome			
Maximal NPIS on POD 0	4.0 (2.0–5.0)	2.0 (2.0–4.0)	0.023
Secondary outcomes			
NPIS at 0 h	2.0 (1.0–3.0)	1.0 (1.0–2.0)	0.092
NPIS at 0.5 h	1.0 (1.0–1.0)	1.0 (1.0–1.0)	0.673
NPIS at 1 h	1.0 (1.0–4.0)	2.0 (1.0–3.0)	0.542
NPIS at 2 h	2.0 (1.0–2.0)	1.0 (1.0–2.0)	0.164
NPIS at 4 h	2.0 (1.0–2.0)	1.0 (1.0–2.0)	0.173
NPIS at 8 h	2.0 (1.0–3.8)	2.0 (1.0–2.0)	0.107
NPIS at 12 h	1.0 (1.0–2.0)	1.0 (1.0–2.0)	0.123
NPIS at 24 h	1.0 (1.0–1.0)	1.0 (1.0–2.0)	0.010
Maximal NPIS on POD 1	1.0 (1.0–1.0)	1.0 (1.0–2.0)	0.018

Data are expressed as median (Q1–Q3). Abbreviation: POD, postoperative day; NPIS, numeric pain intensity scale.

**Table 4:** Pooled adjusted effects of subcutaneous lidocaine infiltration (vs. no infiltration) on postoperative pain outcomes after PSM.

Pain-Related Outcomes	OR (95% CI)	*p*	β (95% CI)	*p*
Primary outcome				
Maximal NPIS on POD 0			−1.25 (−2.01–−0.50)	0.001
Secondary outcomes				
Rescue analgesia on POD 0	0. 12 (0.03–0.46)	0.002		
Number of rescue doses during hospitalization			−1.11 (−1.62–−0.49)	<0.001
Morphine equivalent dose			−5.14 (−7.79–−2.49)	<0.001
NPIS at 0 h			−0.81 (−1.27–−0.35)	<0.001
NPIS at 24 h			0.26 (−0.01–0.53)	0.062
Maximal NPIS on POD 1			0.03 (−0.40–0.46)	0.887

Odds ratios (OR) are presented for binary outcomes, and regression coefficients (β) for continuous outcomes. Only outcomes with *p* < 0.1 in univariate comparisons after PSM were included in the multivariable models. Covariates: age; male sex; BMI ≥ 25 kg/m^2^; ASA PS ≥ III; previous lower abdominal surgery; bilateral hernia; EHS size classification; treatment period; intraoperative IV opioid use; intraoperative IV non-opioid analgesic use; intraoperative IV dexamethasone use; intraoperative IV lidocaine use. The threshold for the secondary NPIS outcomes included in this table (NPIS at 0 h, NPIS at 24 h, and Maximal NPIS on POD 1) was set at *p* < 0.0056 based on Bonferroni correction. Analyses were performed across 20 imputed matched datasets, and regression estimates were pooled using Rubin’s rules. Abbreviations: OR, odds ratio; CI, confidence interval; β, regression coefficient; POD, postoperative day; NPIS, numeric pain intensity scale.

## Data Availability

The data that support the findings of this study are available from the Yongin Severance Hospital Institutional Review Board; however, restrictions apply to the availability of these data, which were used under license for the current study and are not publicly available. However, the data are available from the authors upon reasonable request and with permission from the Yongin Severance Hospital Institutional Review Board.

## Supplementary Materials

The following supporting information can be downloaded at: https://www.mdpi.com/article/10.3390/jcm14238324/s1. Figure S1: Covariate balance before and after propensity score matching, shown for the first imputed matched dataset (representative example).; Table S1: Opioid conversion table for morphine equivalent dose; Table S2: Postoperative pain intensity in PSM cohort expressed as mean ± SD.

## Abbreviations

The following abbreviations are used in this manuscript:

ASA-PS	American Society of Anesthesiologists Physical Status
AUR	Acute Urinary Retention
BMI	Body Mass Index
CI	Confidence Interval
EHS	European Hernia Society
ERAS	Enhanced Recovery After Surgery
HCl	Hydrochloride
IQR	Interquartile Range
IRB	Institutional Review Board
IV	Intravenous
LOS	Length of Stay
NPIS	Numeric Pain Intensity Scale
OR	Odds Ratio
PACU	Post-Anesthesia Care Unit
POD	Postoperative Day
PSM	Propensity Score Matching
QLB	Quadratus Lumborum Block
SD	Standard Deviation
SILTEP	Single-Incision Laparoscopic Totally Extraperitoneal
SMD	Standardized Mean Difference
SSI	Surgical Site Infection
TAP	Transversus Abdominis Plane
TEP	Totally Extraperitoneal
