# Supplementary material for: Single-Shot Subcutaneous Lidocaine Infiltration at Closure Is Associated with Reduced Early Pain and Opioid Requirement After Single-Incision Laparoscopic Totally Extraperitoneal Hernia Repair
Source: J Clin Med. 2025 Nov 23;14(23):8324. doi: 10.3390/jcm14238324 (PMC12692613; doi:10.3390/jcm14238324)

Supplementary Table S1. Opioid conversion table for morphine equivalent dose.

Conversion of Intravenous given drugs to IV morphine equivalents (IME)

Drug	Total daily dose (IV)	Calculate daily IV dose and then multiply by the factor below = IME (mg)
Morphine	mg/day	1
Oxycodone	mg/day	1.5
Hydromorphone	mg/day	4.0
Codeine	mg/day	0.15
Pethidine	mg/day	0.13
Fentanyl	μg/day	0.066
Sufentanil	μg/day	0.66
Parecoxib (Dynastat)	mg/day	0.2 (uncertain/unclear)
Tapentodal	mg/day	0.4
Tramadol	mg/day	0.1
Ketorolac	mg/day	0.4
Transdermal Fentanyl	μg/hour	2.4

Buprenorphine: No conversion factor is available for agonist-antagonist

Conversion of orally taken analgesics to IV morphine equivalents (IME)

Drug	Total daily dose (oral)	Calculate daily oral dose and then multiply by the factor below = IME (mg)
Morphine	mg/day	0.33
Oxycodone	mg/day	0.33
Hydromorphone	mg/day	0.1
Codeine	mg/day	0.25
Pethidine	mg/day	0.4
Fentanyl	μg/day	0.2
Sufentanil	μg/day	2
Tramadol	mg/day	0.033
Tapentadol	mg/day	0.133

Supplementary Table S2. Postoperative pain intensity in the PSM cohort expressed as mean ± SD.

	No lidocaine (n=82)	Lidocaine (n=82)
NPIS at 0h	1.9 ± 1.2	1.5 ± 0.9
NPIS at 0.5h	1.5 ± 1.3	1.3 ± 1.0
NPIS at 1h	2.4 ± 1.8	2.2 ± 1.3
NPIS at 2h	1.9 ± 1.3	1.9 ± 1.4
NPIS at 4h	1.9 ± 1.1	1.6 ± 1.0
NPIS at 8h	2.1 ± 1.4	2.2 ± 1.4
NPIS at 12h	1.3 ± 0.9	1.7 ± 1.1
NPIS at 24h	1.1 ± 0.6	1.3 ± 0.5
Maximal NPIS on POD 0	3.6 ± 1.9	2.8 ± 1.4
Maximal NPIS on POD 1	1.3 ± 1.0	1.5 ± 0.9

Data are expressed as mean ± standard deviation.
NPIS was assessed at 0, 0.5, 1, 2, 4, 8, 12, and 24 hours postoperatively.
Abbreviation: NPIS, numeric pain intensity scale; POD, postoperative day.

Supplementary Figure S1. Covariate balance before and after propensity score matching, shown for the first imputed matched dataset (representative example).

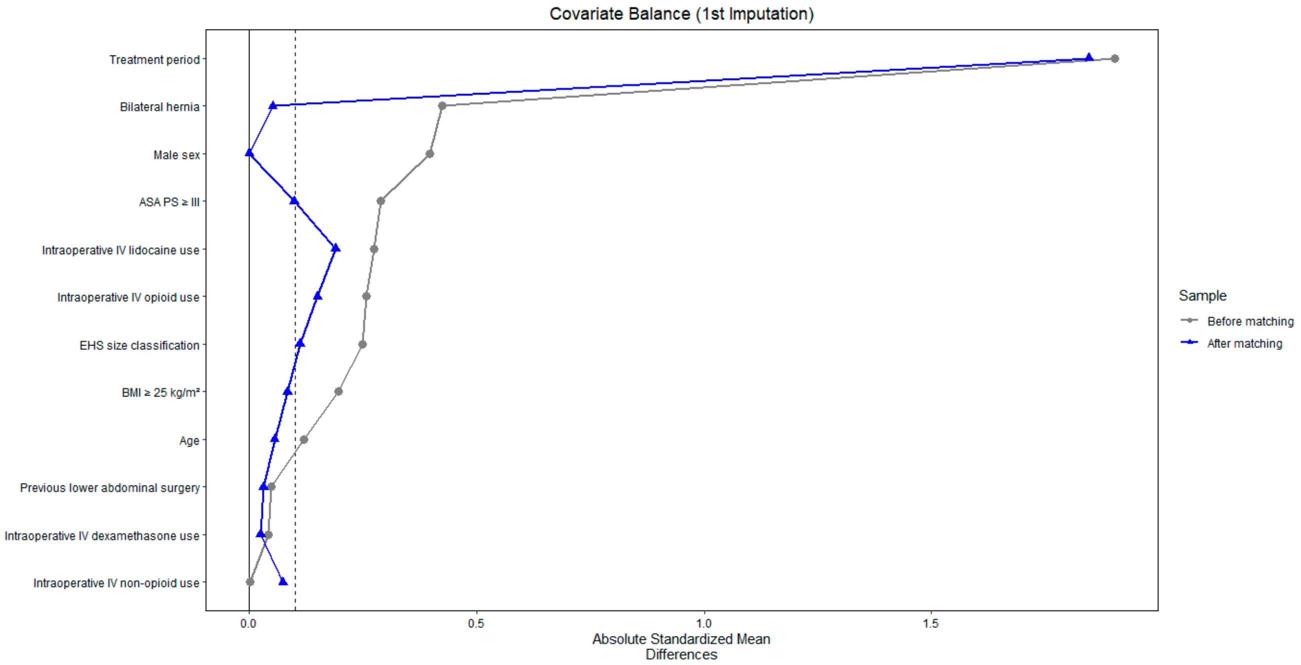

Supplement: Supplementary file 1 [file jcm-14-08324-s001.zip › jcm-3946666-supplementary.pdf]
